# Retraction: α,ß-Didehydrosuberoylanilide hydroxamic acid (DDSAHA) as precursor and possible analogue of the anticancer drug SAHA

**DOI:** 10.3762/bjoc.22.86

**Published:** 2026-07-21

**Authors:** Shital K Chattopadhyay, Subhankar Ghosh, Sarita Sarkar, Kakali Bhadra

**Affiliations:** 1 Department of Chemistry, University of Kalyani, Kalyani - 741235, West Bengal, India; 2 Department of Zoology, University of Kalyani, Kalyani - 741235, West Bengal, India

**Keywords:** anticancer drug, cross metathesis, HDAC inhibition, hydroxamates, reactive oxygen species

This article has been retracted due to suspected, improper image manipulation and duplication. Following a report of discrepancies in biological data by a third party on PubPeer [1], an investigation confirmed that Figure 6A likely contains manipulated and duplicated content. Specifically, Figure 6A was found to be an edited version of Figure 8A in *J. Mol. Recognit.* **2018,** *31,* No. e2687 (https://doi.org/10.1002/jmr.2687). Additionally, the middle panel of Figure 6A was mostly a duplicate of the middle panel of Figure 2C in *J. Appl. Biol. Biotechnol.* **2018,** *6,* 1–8 (https://doi.org/10.7324/JABB.2018.60401). According to the original publication, these panels were presented as representing different experimental contexts. The similarity between the images, however, indicated that the figures do not accurately reflect distinct experimental data.

The authors of the original publication responded to the investigation and denied any evidence of data fabrication or misconduct. They stated that the figures depict different cell types and differ in both their biological characteristics and morphometric features. The authors further argued that in the original uncompressed graphics, pixel-level differences would be evident between the corresponding images. However, upon request they did not provide the original uncompressed images to support this assertion, stating that they were no longer available due to limitations in data retention.

## References

[1] (2026). Comment by Sundasciurus fraterculus on “Binding affinity and in vitro cytotoxicity of harmaline targeting different motifs of nucleic acids: An ultimate drug designing approach”, September 2025.

